# Solving a microplastic dilemma? Evaluating additive release with a dynamic leaching method for microplastic assessment (DyLeMMA)

**DOI:** 10.1016/j.mex.2023.102221

**Published:** 2023-05-19

**Authors:** James H. Bridson, Robert Abbel, Dawn A. Smith, Grant L. Northcott, Sally Gaw

**Affiliations:** a Scion, Titokorangi Drive, Private Bag 3020, Rotorua 3046, New Zealand; b School of Physical and Chemical Sciences, University of Canterbury, Christchurch 8041, New Zealand; c Northcott Research Consultants Limited, 20 River Oaks Place, Hamilton 3200, New Zealand

**Keywords:** Plastic pollution, Additive, Non-intentionally added substance, Intentionally added substance, Extractable, Leachable, Release kinetics, **DyLeMMA**: **Dy**namic **Le**aching **M**ethod for **M**icroplastic **A**ssessment

## Abstract

Microplastics and plastic additives are contaminants of emerging environmental concern. Static leaching methods are commonly applied to assess the rate and extent of additive release from microplastics. However, this approach may not be representative of environmental conditions where near infinite dilution or percolation commonly occur. We evaluated three different approaches for assessing additive leaching under environmentally relevant sink conditions, culminating in the refinement and validation of **DyLeMMA** (**Dy**namic **Le**aching **M**ethod for **M**icroplastic **A**ssessment). Analysis was performed using a high-resolution liquid chromatography-mass spectrometry method enabling targeted quantification of additives and screening for non-intentionally added substances. Using four different plastics, sink conditions were maintained over the duration of the test, thereby avoiding solubility limited release and ensuring environmental relevance. Background contamination from ubiquitous additive chemicals was minimised, thereby providing good sensitivity and specificity. Resulting data, in the form of additive release curves, should prove suitable for fitting to release models and derivation of parameters describing additive leaching from microplastics.Key attributes of DyLeMMA:

• Environmentally relevant dynamic leaching method for microplastics, demonstrated to maintain sink conditions over the test duration,
• Simple, fast, and cost-effective approach without complication of using a solid phase sink,
• Provide data suitable for understanding microplastic leaching kinetics and mechanisms.

## Graphical abstract

**Figure:**
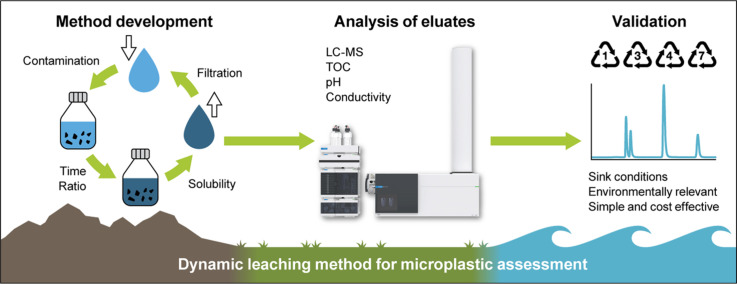

Specifications table

**Table:** 

Subject Area:	Environmental Science
More specific subject area:	Microplastic pollution
Name of your method:	**DyLeMMA**: **Dy**namic **Le**aching **M**ethod for **M**icroplastic **A**ssessment
Name and reference of original method:	CEN/TS 16637–2:2014 Construction products: Assessment of release of dangerous substances – Part 2: Horizontal dynamic surface leaching test.
Resource availability:	Shaking incubator (Lab Companion, IST-4075) Centrifugal evaporator (SP Genevac EZ-2 Plus) and nitrogen blowdown apparatus LC-MS instrument (Agilent 1290 Infinity II UHPLC system with Agilent 6545 AdvanceBio QTOF mass spectrometer) and data processing software (Agilent MassHunter 10.0) TOC analyser (GE Sievers InnovOx Laboratory TOC Analyser)

## Method details

### Background information

Plastics contain additives which are incorporated into base polymers during manufacturing to impart beneficial properties to the material such as colour, flexibility, or heat stability. In addition, plastics may also contain residual substances from the synthesis process such as monomers, solvents, and catalysts [1]. Non-intentionally added substances (NIAS) are chemicals in plastics that arise from degradation, contamination, or impurities which accumulate during manufacturing or use. Most additives, residual substances, and NIAS can leach from the plastic into the surrounding environment over time because they are not chemically bound to the polymer matrix [2]. In the fields of food and pharmaceutical packaging the leaching of additives and NIAS from plastic is well recognised and this has resulted in well-established and standardised methods to evaluate their leaching from plastics [3]. However, studies examining the leaching of additives from plastic pollution in an environmental context are comparatively recent. Furthermore, a wide range of disparate experimental approaches have been used to evaluate the leaching of additives from plastic pollution, often without consideration of environmental relevance. Progress towards the development of standardised methods in the field of microplastic research has been identified as a critical step to advance efforts in understanding the impact of microplastic pollution, developing sustainable solutions, and implementing regulation [4,5,6].

The most common methods used to evaluate leaching of additives from plastic pollution employ a static approach, leading to equilibrium partitioning between the plastic sample and aqueous phase [7,8,9,10]. This may be appropriate for food or pharmaceutical packaging scenarios where the media (packaged food item) is static, but it is not representative of environmental conditions where dilution or percolation occur, such as in rivers and streams, the open ocean, or soil environments. In these situations, a more environmentally relevant approach is the use of dynamic leaching methods, where the analyte concentration is maintained well below the solubility limit (sink conditions) and equilibrium partitioning of released additives in not achieved. Dynamic conditions for plastic additive leaching have been achieved using column percolation [11], an automated flow-based system [12], or a solid phase infinite sink such as activated carbon [13,14,15]. Alternatively, dynamic conditions can be achieved by periodic replenishment of the media as used in European Committee for Standardization Technical Specification 16637–2:2014 for assessment of construction products [16] or American Standard Test Method D4793 for the assessment of waste [17].

We report the development and validation of a new Dynamic Leaching Method for Microplastics Assessment (DyLeMMA) that evaluates additive release from microplastics under realistic environmental conditions. In this method eluates are analysed using UHPLC-QTOF-MS to detect and quantify target additives and identify NIAS. The leaching method was demonstrated using four plastics (polyethylene, polyethylene terephthalate, nylon 6, and polyvinyl chloride) compounded with six commonly used additives of several classes (phthalate, phenolic benzotriazole, hindered phenolic antioxidant, organophosphite, and hindered amine stabiliser) covering a diverse range of physicochemical properties.

### Reagents and standards

LC-MS grade solvents methanol (≥ 99.97%, CAS 67–56–1) and water (LiChrosolv®, CAS 7732–18–5), sourced from Merck, and formic acid (≥ 99.0%, CAS 64–18–6), sourced from ThermoFisher, were used as received. Ultrapure (UP) water (ASTM type 1) was generated using a Sartorius Arium® pro DI/UV (18.2 MΩcm) water purification system. All other solvents were of analytical grade sourced from Merck and used as received. The analytical reference materials octadecyl 3-(3,5-di‑tert‑butyl‑4-hydroxyphenyl) propionate (AO-1076, 99%, CAS 2082–79–3), pentaerythritol tetrakis(3-(3,5-di‑tert‑butyl‑4-hydroxyphenyl) propionate) (AO-1010, 98%, CAS 6683–19–8), tris(2,4-di‑tert-butylphenyl) phosphite (AO-168, 98%, CAS 31570–04–4), 2-(2H-benzotriazol-2-yl)−4,6-bis(1-methyl-1-phenylethyl) phenol (UV-234, 98%, CAS 70321–86–7), and dibutyl phthalate (DBP, 99%, CAS 84–74–2) were sourced from Sigma Aldrich and N,N'-Bis(2,2,6,6-tetramethyl-4-piperidyl)isophthalamide (HAS-1, 96%, CAS 42774–15–2) from Tokyo Chemical Industry. The isotopically labelled standards 2-(2H-benzotriazol-2-yl)−4,6-bis(1-methyl-1-phenylethyl) phenol-d_4_ (UV-234-d_4_) and bis(2,2,6,6-tetramethyl-4-piperidyl) sebacate-d_24_ (HAS-2-d_24_) were sourced from ASCA GmbH, octadecyl 3-(3,5-di‑tert‑butyl‑4-hydroxyphenyl) propionate-d_37_ (AO-1076-d_37_) from Toronto Research Chemicals, and dibutyl phthalate-d_4_ (DBP-d_4_) from Sigma Aldrich. Solid phase extraction discs (Affinisep AttractSPE® HLB, C18, and SDB-RPS) were sourced from PM Separations.

### Microplastic sample preparation

Plastic samples for method evaluation and validation were prepared as described previously [18]. Briefly, polymer resins were compounded with common additives at proportions representative of those typically used in industry and compression moulded into sheets of 1.2 mm nominal thickness. The plastic composition is described in Table 1. The compression moulded plastic sheets were cut by hand into microplastic sized pieces (2 × 2 mm) using shears, wrapped in aluminium foil, and stored at room temperature in dark condition before use.

**Table 1:** Details of samples used for method evaluation and validation.

Sample	Polymer	Additive (concentration (w/w))
PA6_HAS	Nylon 6	HAS-1 (0.41%)
PET_UV	Polyethylene terephthalate	UV-234 (0.22%)
PVC_DBP	Polyvinyl chloride	DBP (21%)
LDPE_AO	Low density polyethylene	AO-1076 (0.14%), AO-1010 (0.05%), AO-168 (0.15%)

### Dynamic leaching method for microplastic assessment

The DyLeMMA was based on CEN/TS 16637–2:2014 for the assessment of release of dangerous substances from construction products [16]. Microplastic samples were immersed in UP water (media) and incubated using a Lab Companion IST-4075 incubated shaker for up to 64 d. The detailed DyLeMMA procedure was as follows:

1. Accurately weigh 500 mg of microplastic sample (25 mg of PVC_DBP) into a 50 mL amber borosilicate glass bottle. Prepare three replicate bottles for each microplastic sample.
2. To start the leaching experiment, consecutively add 50 mL of UP water to each bottle with a 2 min interval between bottles. Close bottles using a PTFE lined cap.
3. Place bottles vertically into an incubated shaker set at 30 °C with orbital shaking at 120 rpm under dark conditions.
4. At each sampling time point (Table 2), consecutively remove bottles from the incubator.

**Table 2:** DyLeMMA sampling time points.

Step	Duration from test start	Duration from previous step	Example planning of sampling time points
Start	0 h	N/A	Week 1 Monday 9 am
1	6 h	6 h	Week 1 Monday 3 pm
2	1 d	18 h	Week 1 Tuesday 9 am
3	2.25 d	1.25 d	Week 1 Wednesday 3 pm
4	4 d	1.75 d	Week 1 Friday 9 am
5	9 d	5 d	Week 2 Wednesday 9 am
6	16 d	7 d	Week 3 Wednesday 9 am
7	36 d	20 d	Week 6 Tuesday 9 am
8	64 d	28 d	Week 10 Tuesday 9 am
5. Decant the entire water phase (eluate) through a stainless steel wire filter cloth (100 mesh), folded to sit inside a glass funnel, into a 60 mL amber glass vial.
6. Using forceps carefully retrieve any plastic pieces from the wire filter cloth and return to the bottle.
7. Add 50 mL of fresh UP water to each bottle, close, and return to the incubator
8. Seal 60 mL amber glass vials using a PTFE lined cap and store in the dark at 4 °C before analysis.

### Eluate analysis

Eluate pre-concentration and solvent exchange were performed by evaporation using either a SP Genevac EZ-2 Plus centrifugal evaporator at 30 °C under a nitrogen purge (aqueous program with automatic end detection), or a blowdown apparatus under a nitrogen purge at 30 °C (PVC_DBP samples). In accordance with the level of pre-concentration required, 0.1 to 15 mL of eluate was transferred into a 2 mL high recovery micro V vial (Agilent) or 15 mL glass digestion tube with 10 or 100 µL of internal standard and evaporated to dryness. Eluates prepared in 15 mL glass digestion tubes were transferred into high recovery micro V vials using 3 × 400 µL aliquots of acetone + 0.1% formic acid and evaporated to dryness as above. Vial contents were re-dissolved in 100 µL of methanol (or 1000 µL of 1:1 methanol/water (PA6_HAS and PVC_DBP samples)) and analysed within 24 h.

Eluates were analysed using an Agilent 1290 Infinity II UHPLC system with an Agilent 6545 AdvanceBio quadrupole time-of-flight mass spectrometer and Agilent Jet Stream electrospray ionisation source. Chromatographic separation was performed using an analytical column (Agilent Zorbax RRHD Eclipse Plus C18, 2.1 × 50 mm, 1.8 µm) and equivalent guard column (2.1 × 5 mm) eluted at 0.4 mL/min at a column temperature of 40 °C. The solvents used as mobile phase were 10% methanol in water (mobile phase A) and methanol (mobile phase B), both with 0.1% formic acid. The gradient was initiated at 5% mobile phase B and held isocratic for 1 min before increasing to 100% mobile phase B from 1 to 3 min and held isocratic until completion of the run at 8 min. The volume of injected sample was 5 µL. Detection was performed in positive ion mode using typical source settings (de-solvation gas (300 °C at 12 L/min), nebuliser pressure (35 psi), and sheath gas (275 °C at 12 L/min)) and optimised MS parameters (capillary voltage (4000 V), nozzle voltage (500 V), and fragmentor voltage (150 V)). Instrument calibration and tuning was performed by direct infusion of a calibrant mixture (Agilent ESI-L low concentration tuning mix). Data were collected using standard mass range (100 to 3000 *m/z*) in high sensitivity extended dynamic range mode to obtain full scan high resolution data at a mass resolution of 30,000 full width at half maximum (*m/z* 450). Data was processed using Agilent MassHunter Quantitative 10.0 software, with the identity of target compounds (Table 3) confirmed based on accurate mass (± 10 ppm), retention time (± 0.5 min), and carbon isotope ratio. For each batch of analysed samples, a stock calibration standard and internal verification standard were freshly prepared containing all target analytes at 1 mg/mL in acetone. Stock standards were diluted to 0.1 to 1000 ng/mL in methanol. An internal standard, containing all four isotopically labelled internal standards, was prepared at approximately 10 µg/mL and added to samples and standards at 10 ng/mL for instrumental analysis. Non-target compounds were identified using Agilent MassHunter Qualitative 10.0 software using the target/suspect screening Find by Formula workflow. Chromatographic peaks were compared with the Agilent Extractables and Leachables Personal Compound Database and Library (PCDL) or an in-house monomers and oligomers PCDL to provide tentative identification.

**Table 3:** Molecular formula, CAS, and LC-MS parameters of target analytes.

Analyte	CAS	Molecular formula	R_T_ (min)	Monoisotopic mass	Ion *m/z*	Species	IS
HAS-1	42774–15–2	C_26_H_42_N_4_O_2_	2.2	442.3308	222.1729	[M + H]^2+^	a
HAS-2-d_24_ (a)	NA	C_28_H_28_D_24_O_4_N_2_	2.8	504.5434	253.2798	[M + H]^2+^	
DBP	84–74–2	C_16_H_22_O_4_	3.7	278.1518	301.1410	[M+Na]^+^	b
DBP-d_4_ (b)	93952–11–5	C_16_H_18_D_4_O_4_	3.7	282.1769	305.1661	[M+Na]^+^	
UV-234	70321–86–7	C_30_H_29_N_3_O	4.5	447.2311	448.2383	[M + H]^+^	c
UV-234-d_4_ (c)	NA	C_30_H_25_D_4_N_3_O	4.5	451.2562	452.2634	[M + H]^+^	
AO-1010	6683–19–8	C_73_H_108_O_12_	4.8	1176.784	1199.7733	[M+Na]^+^	d
AO-1076-d_37_ (d)	NA	C_35_H_25_D_37_O_3_	5.9	567.7021	590.6914	[M+Na]^+^	
AO-1076	2082–79–3	C_35_H_62_O_3_	6.0	530.4699	553.4591	[M+Na]^+^	d
AO-168	31570–04–4	C_42_H_63_O_3_P	7.2	646.4515	647.4588	[M + H]^+^	d

R_T_ = retention time; IS = internal standard used for quantification.

Non-specific analyses were performed without any eluate preparation steps. Conductivity was measured using a Thermo Scientific Eutech COND 6+ conductivity metre and pH was measured using a Denver Instruments UB-10 metre. Non-purgeable organic carbon (NPOC) was determined using a GE Sievers InnovOx laboratory TOC analyser with GE autosampler using sodium persulfate and perchloric acid reagents. Eluates were analysed with 15% oxidiser, 1% acid, sparge time of 0.8 min, single sample flush, and blank correction with three replicate measurements per eluate. Calibration was performed using a freshly prepared potassium hydrogen phthalate standard.

### Method validation

Validation of the DyLeMMA was performed using different polymer resins, plastic additives, and plastic (solid) to water (liquid) ratios, with recovery and reproducibility also assessed. Four different plastic formulations were evaluated covering four polymers (LDPE, PA6, PET, and PVC) and six additives (AO-1076, AO-1010, AO-168, UV-234, HAS-1 and DBP). For each plastic, the method was evaluated at three different solid to liquid ratios. For LDPE_AO, PET_UV, and PA6_HAS solid to liquid ratios of 250 mg (low), 500 mg (mid), and 1000 mg (high) per 50 mL were evaluated. For PVC_DBP solid to liquid ratios of 10 mg (low), 25 mg (mid), and 250 mg (high) per 50 mL were evaluated. Recovery was determined by mass balance, with microplastic samples recovered at the conclusion of the leaching experiment, dried (40 °C under vacuum for 72 h), weighed, and solvent extracted. Solvent extraction was performed by accurately weighing 100 mg of PA6_HAS or 10 mg of PVD_DBP into an extraction tube, adding 4 mL of solvent (methanol for PA6_HAS and diethyl ether for PVC_DBP), and sonicating (Bandelin SONOREX) for 30 min. The extraction process was repeated three times with fresh solvent. The extracts were combined in a volumetric flask, made to volume with extraction solvent, and analysed by LC-MS. Reproducibility of the DyLeMMA was assessed using replicate leaching treatments (*n* = 3).

The LC-MS and NPOC methods were validated according to Eurachem guidelines [19] with respect to specificity, limit of detection (LOD), limit of quantification (LOQ), linearity, range, accuracy, and precision (repeatability) (Table 4). For the LC-MS method, specificity was evaluated comparing chromatograms of five replicate matrix (UP water) blanks, diluent blanks, and zero calibration blanks with matrix spikes. The LOD and LOQ were calculated as three or ten times the standard deviation, respectively, from ten replicate injections near the lowest detectable concentration of each target analyte according to EPA guidelines [20]. Linearity and range were examined with 13 calibration levels over a concentration range of 0.1 to 1000 ng/mL with three replicates at each level. Homoscedasticity was tested using an F-test (α 0.05) and goodness of fit quantified by correlation coefficient (R^2^) and percent residual accuracy (%RA) [21]. Accuracy and precision were determined by spiking leaching media at concentrations ranging from 0.05 to 500 ng/mL, as relevant for each analyte, with ten replicates at each level. Precision was assessed as percent relative standard deviation (RSD) under repeatability conditions. For the NPOC method, specificity was determined using UP water. The LOD and LOQ were determined from ten replicate analyses of near zero concentration of potassium hydrogen phthalate (allowing for blank correction), calculated as three or ten times *s’_o_*, respectively, as follows:

$$s_{o}^{{}^{\prime }}=s_{o}\sqrt{\frac{1}{n}+\frac{1}{n_{b}}}$$

Where s_o_ is the estimated standard deviation of near zero concentration; n is the number of replicate observations; and n_b_ is the number of blank observations. Linearity and range were determined with five calibration levels from 0 to 100 ppm. Accuracy and precision were determined from ten replicate analyses of UP water spiked at 25 ppm potassium hydrogen phthalate.

**Table 4:** Summary of the quantitative LC-MS method performance and validation results.

Analyte	Linearity and range				Sensitivity (ng/mL)		Accuracy (%) at spike level (ng/mL)					Precision (%RSD) at spike level (ng/mL)				
	Range (ng/mL)	Weight	R^2^	%RA	LOD	LOQ	500	50	5	0.5	0.05	500	50	5	0.5	0.05
HAS-1	5–1000	1/x	0.995	90.8	1.22	4.06	102.4	95.0	–	–	–	1.5	3.5	–	–	–
DBP	5–1000	1/x	0.995	91.6	1.27	4.22	91.8	86.2	–	–	–	6.0	4.3	–	–	–
UV-234	0.1–100	1/x	0.995	90.3	0.03	0.09	–	–	98.2	108.9	112.9	–	–	3.7	6.8	4.6
AO-1010	0.1–100	1/x	0.996	91.6	0.02	0.07	–	–	105.1	109.4	97.9	–	–	3.9	9.1	13.5
AO-1076	0.25–100	1/x	0.996	91.8	0.05	0.16	–	–	95.9	117.8	168.0	–	–	2.1	5.8	13.5
AO-168	0.25–250	1/x	0.998	90.6	0.03	0.11	–	–	67.3	6.2	3.0	–	–	3.4	108.0	131.0

R^2^ = correlation coefficient;%RA = percent residual accuracy; LOD = limit of detection; LOQ = limit of quantification;%RSD = percent residual standard deviation.

### Quality assurance

To minimise contamination from plastic additives and other organic substances that could interfere in the analysis of target compounds or be mistakenly identified as NIAS, all glassware was rinsed three times with propan-2-ol, acetone, and UP water before heating to 450 °C for 6 h before use. Non-glassware items were thoroughly cleaned with acetone before use (e.g. scissors, spatula, forceps). Plastic samples were always handled with gloves and where possible aluminium trays or foil were used to hold and store samples.

## Additional information

### Development of DyLeMMA

The objective of this work was to identify an environmentally relevant leaching method to determine the release of additives per unit mass as a function of time for microplastics. Three different dynamic leaching approaches were initially evaluated for the ability to provide sink conditions, these being: (i) an immiscible organic solvent sink, (ii) a solid phase sink, and (iii) sequential media replenishment (Method S1). The approach using an immiscible organic solvent sink was based on a dissolution testing method used in the pharmaceutical industry for drugs of low water solubility [22]. n-Octanol has been commonly used for this purpose due to its immiscibility with water, density less than that of water, and widespread use in determining partition coefficients for many organic compounds. The solid phase sink approach employed solid phase extraction (SPE) discs as a sink substrate. This was based on similar methods described in the literature using activated carbon [13] or polyethylene [14,15]. However, these solid phase sink methods present significant challenges, with activated carbon being difficult to separate and recover from the media and polyethylene potentially introducing plastic additive contaminants. Polymeric sinks are also commonly used for studying the release of environmental contaminants from soil and sediments. Commonly used materials include Amberlite® XAD® (crosslinked polystyrene copolymer), Tenax® (poly(2,6-diphenyl-p-phenylene oxide)), silicones, and polyethylene [23,24]. Again, consideration must be given to these polymeric materials being a source of additives or other contaminates. SPE discs were proposed as an alternative sink material providing a high surface area and capacity, tuneable chemistry, and high purity to minimise contamination. Lastly, a sequential batch leaching method was evaluated. This approach is employed in the standardised testing of leaching from construction materials and wastes [17,16]. With regards to plastic pollution, this experimental approach has been previously used to determine the release of additives from microfibres [25] and consumer plastic items [26]. However, in both prior studies no consideration to assessing sink conditions was made. Sink conditions can be maintained if the analyte concentration remains below the additive water solubility limit, and can be assured by adopting the recommended threshold of one third the solubility limit as applied in pharmaceutical testing of poorly water soluble drugs [22].

To assess the suitability of the sink methods, experiments were first performed using aqueous solutions spiked with additives. Using octanol as an immiscible sink, partitioning occurred very rapidly with near complete transfer from the aqueous phase to the octanol phase occurring within 22 h (Fig. S1). Partitioning to SPE discs was considerably slower, with incomplete transfer after 4 d (Fig. S2). Of the three different SPE substrates evaluated, the HLB adsorbent phase exhibited greatest uptake of AO-1076 and UV-234 and was therefore used for further experiments herein. Proof-of-concept leaching experiments using all three approaches were performed using LDPE_AO and PET_UV plastics for up to 16 d. Persistent contamination of n-octanol with the target analytes AO-1076 and AO-1010 and an inability to achieve analyte concentration (due to high boiling point of n-octanol) led to poor sensitivity of the immiscible sink approach. Sensitivity was greatly improved using the SPE sink approach due to the inherent concentration of analytes onto the SPE disc and minimal background contamination. But this approach was time consuming and expensive due to the labour-intensive back extraction of analytes and costly consumable requirements. For the sequential batch leaching approach, dynamic conditions were evaluated by comparing the analyte concentrations to solubility limits (Table S1). For all analytes evaluated, the maximum observed concentration of additive leached into the aqueous solution was less than 50% of the solubility limit indicating that sink conditions were maintained at each sampling point. Comparing the three different dynamic leaching approaches, the sequential batch leaching method was selected for further studies, owing to minimal background contamination, adequate sensitivity, cost effectiveness, and simplicity (Table S2). This approach produced a suitable volume of leachate to enable other non-specific analyses to be performed on the eluates including measuring pH, conductivity, and NPOC, which are not possible with the n-octanol and SPE disc sink methods.

The sequential batch leaching method was further developed with respect to optimising sampling time points, solid to liquid ratio, and sample handling. Sampling time points were selected as prescribed in CEN/TS 16637–2:2014 (Table 2), being designed such that the concentration of additive leached into the aqueous solution (media) should not exceed its water solubility limit and thereby adversely affect the release rate [16]. The test specimens (microplastics) were placed in a sealed glass bottle and immersed in water (media) of a given volume at a specific solid to liquid ratio and incubated at a constant temperature (30 °C). At predetermined time intervals the water was decanted and renewed, and the recovered solution (eluate) subsequently characterised to quantify the concentration of additives. Using the procedure other non-specific methods of analysis could also be applied to characterise the eluate such as pH, conductivity, and NPOC to provide supporting information on the leaching process. During the sampling step, filtration of the eluate was desired to remove plastic fragments that could contaminate the sample leading to erroneous results. The use of syringe filters and filter membranes was initially evaluated, however poor recover of the more hydrophobic analytes (AO-1076, AO-1010, A)−168, and UV-234) was observed (Table S3). This was attributed to analyte adsorption to the membrane or housing as previously demonstrated for plastic leachables [27]. Furthermore, polymeric filtration membranes may lead to contamination of samples through the leaching of hydrophilic compounds during use [28]. As such, a stainless steel wire filter cloth was selected rather than polymeric filters to minimise potential contamination and analyte adsorption. A liquid to surface area ratio is prescribed in CEN/TS 16637–2:2014, however for microplastics accurately determining the surface area can be difficult. Therefore, a mass to liquid ratio was applied enabling more precise gravimetric determination of the quantity of plastic in each leaching vessel. The mass of plastic and volume of media were dimensioned based on the proportion of additives in the experimental plastics, expected release rates from previously published work, and sensitivity of additive quantification. Stabiliser additives are commonly incorporated into plastics at 0.1 to 3% (w/w), while plasticisers may be used at up to 70% (w/w) [1,2], with cumulative release over experimental timeframes spanning several orders of magnitude [26]. To this end, a solid to liquid ratio of 1% (w/v) (500 mg of microplastic per 50 mL water) was employed for samples PA6_HAS, PET_UV, and LDPE_AO. The solid to liquid ratio was reduced to 0.05% (w/v) (25 mg of microplastic per 50 mL water) for PVC_DBP to accommodate the significantly higher proportion of additive in the plastic and the relatively higher water solubility of DBP compared to the other target additives.

For analysis of organic compounds, consideration should be given to the potential for abiotic and biotic transformation of analytes. The use of a preservative such as sodium azide is recommended in CEN/TS 16637–2:2014 to inhibit microbial activity and biodegradation of study chemicals for the duration of the test procedure. However, the hazardous nature of sodium azide may complicate subsequent analysis of the eluates. As such, no preservative was used in this study, however all practicable steps were taken to minimise biological growth including sterilisation of glass bottles (muffle oven), use of sterile UP water, minimising head space, and exclusion of light (using amber glass bottle and enclosing incubator in aluminium foil).

A plastic sampling approach was developed to ensure representative sampling and appropriate handling to minimise sample deterioration, contamination, and bias. Before analysis the microplastic samples were stored wrapped in aluminium foil to avoid contamination from plastic packaging and also to exclude light which may lead to polymer degradation. Three replicate samples (compression moulded sheets) were size reduced to microplastic pieces by hand cutting with shears. This approach was chosen to minimise cross contamination, avoid creating new surfaces or changes to the surface morphology of the moulded plastic sheets, and provide a more homogenous size distribution of microplastic pieces. Alternatively, grinding and sieving could be applied depending upon the study aim and hypotheses, however consideration should be given to the uniformity of particle size. During sample preparation care should be taken to avoid heat generation, for example during milling, which may degrade or change the physicochemical properties of the plastic and lead to consumption of stabiliser additives.

### LC-MS method development

Functional organic plastic additives are commonly hydrophobic non-volatile compounds, ideally suited to analysis by LC based methods. The primary requirements of a quantitative method to evaluate additive leaching include short run time, high sensitivity, and wide dynamic range. Additionally, the ability to detect non-target analytes is also highly desirable to study the leaching of NIAS and residual substances. As such, a UHPLC-QTOF-MS based method was chosen as the best approach to fulfil these requirements [29]. With guidance from previously published methods [30,31] an initial method was developed and optimised with respect to the LC (mobile phase, gradient, injection volume, and column temperature) and MS (capillary voltage, nozzle voltage, and fragmentor voltage) operating parameters. Methanol was selected as mobile phase in preference to acetonitrile as it provided greater sensitivity for the target additives, reduced run time, improved peak shape, and fewer background contaminants. A steep gradient provided adequate retention of the less hydrophobic analytes (HAS-1), while minimising run time (< 10 min). A large injection volume (5 µL) in methanol was used to enhance sensitivity and to ensure solubility of the more hydrophobic analytes. However, this led to splitting of the HAS-1 peak. This was overcome by increasing the polarity of the injection solvent (1:1 methanol/water) for HAS-1 analysis. Increasing the column temperature from 30 to 40 °C decreased the retention time of the late eluting peaks (AO-1076 and AO-168) by ca. 5% but further increases in column temperature had a negligible impact on retention time. The MS interface conditions were optimised to enhance the detection sensitivity (Table S4), with a compromise in settings selected to favour the more hydrophobic analytes expected to be at the lowest concentrations in the eluates (e.g. AO-1010, UV-234).

### Analytical method validation

The UHPLC-QTOF-MS method and sample preparation were validated with respect to specificity, LOD, LOQ, linearity, range, accuracy, and precision (Table 4).

Specificity of the method was demonstrated by comparing blanks with spiked samples. Solvent blank injections exhibited DBP and AO-1076 ghost peaks which were attributed to contamination of the solvents used to prepare the mobile phase. Matrix blanks (UP water) exhibited AO-1010 at concentrations near the LOQ, attributed to acetone used during sample preparation. While steps were taken to minimise background contamination of these additives (see quality assurance discussion), they could not be fully eliminated, indicating the ubiquity of these compounds. All other target compounds were absent in the blanks.

The LOD and LOQ for the six target analytes ranged from 0.02 to 1.27 ng/mL and 0.07 to 4.22 ng/mL, respectively. The sensitivity was an order of magnitude greater for the more hydrophobic analytes (AO-1076, AO-1010, AO-168 and UV-234) compared with DBP and HAS-1. For DBP, the comparatively poorer sensitivity was attributed to the persistent background contribution and resulting presence in all sample chromatograms. For HAS-1 ionisation efficiency was compromised by operating with nozzle and fragmentor voltages set to favour sensitivity of the more hydrophobic compounds, which were expected to be at lower concentration in leachates. Despite these limitations the sensitivity obtained by our method compared favourably with previously reported LC-MS methods. By comparison, LOD values of 0.98 ng/mL for AO-1076, 1.84 ng/mL for AO-1010, and 25 ng/mL for DBP were achieved using a LC-MS/MS method in multiple reaction monitoring mode [32,33], and LOD values of 0.2 ng/mL for AO-1076, 1.0 ng/mL for AO-1010 and 0.3 ng/mL for AO-168 were obtained using a high resolution LC-QTOF in full scan mode [30].

Linearity was evaluated from the LOQ up to 1000 ng/mL concentration (*n* ≥ 8) to determine the working range and appropriate curve fit. Heteroscedasticity was confirmed for all analytes, which combined with the wide dynamic range, necessitated the evaluation of weighted least squares regression (Table S5). For all analytes a 1/x weighting was selected based on visual analysis of the calibration curves and application of goodness of fit tests. Goodness of fit was demonstrated using both correlation coefficient and percent residual accuracy, with all calibration curves exhibiting R^2^ ≥ 0.995 and %RA ≥ 90%. This resulted in a linear dynamic range of two to three orders of magnitude.

Accuracy and precision were evaluated at concentrations relevant for each analyte. As such, poorly water-soluble analytes AO-1076, AO-1010, AO-168, and UV-234 (water solubility < 100 ng/mL) were spiked at concentrations of 0.05, 0.5, and 5 ng/mL to determine accuracy and precision. By contrast, the more water-soluble additives HAS-1 and DBP were spiked at 50 and 500 ng/mL for determination of accuracy and precision. HAS-1 and DBP accuracy was within ± 15% with repeatability ≤ 6% RSD at both spike concentrations. AO-1076, AO-1010, and UV-234 accuracy was within ± 5% with repeatability ≤ 4% RSD at the highest spike concentration, however accuracy and precision for AO-1076 deteriorated at lower concentrations. The accuracy of AO-168 was low with poor repeatability at spike concentrations ≤ 0.5 ng/mL, which was attributed to degradation during sample preparation, as previously observed [34].

NPOC method validation was performed with respect to specificity, LOD, LOQ, linearity, range, carryover, accuracy, and precision (Table S6). The method range of 0 to 100 ppm was suitable for all eluates with concentrations of NPOC in eluates typically less than 50 ppm. The LOD was improved by baking of autosampler vials at 450 °C to remove traces of organic matter. Accuracy and precision were acceptable for this method.

### Validation of DyLeMMA

Specificity of the DyLeMMA was demonstrated using UP water incubated alongside plastic leaching samples. All additives were below the LOD in blank samples, except AO-1010 as detailed above. The concentration of AO-1010 in all blank samples was reasonably consistent at approximately 0.001 ng/mL. Minimal co-eluting non-target compounds were observed in the total ion chromatograms of the UP water control treatment, which could lead to interference and ion suppression of the target additive chemicals.

**Fig. 1:**
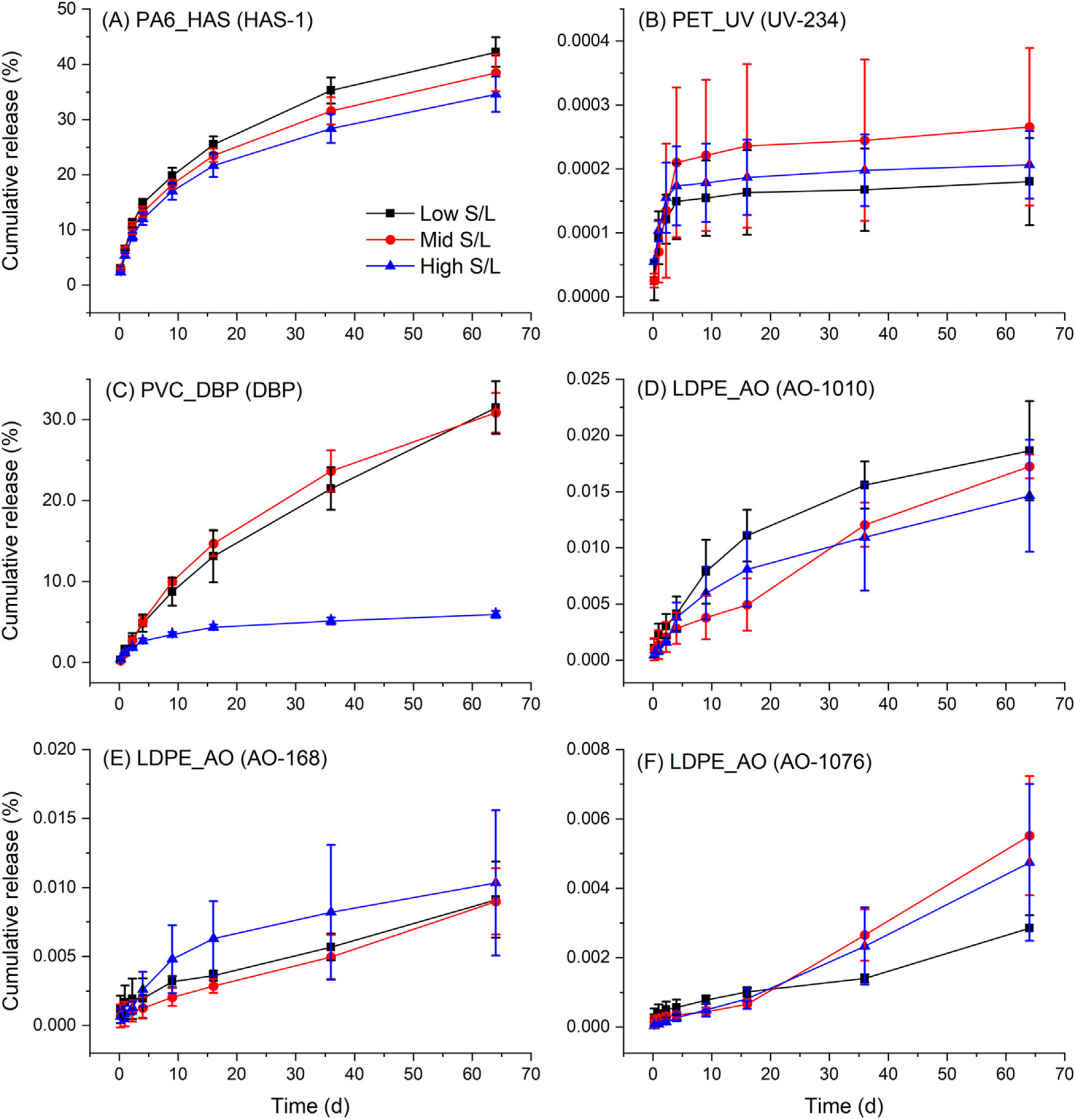
Cumulative additive release profiles (as a percent of additive mass) demonstrated for all plastic-additive combinations at a low, mid, and high solid to liquid ratio (error bars = standard deviation).

To evaluate if sink conditions were maintained, leaching experiments were performed at different plastic to liquid ratios. In the case that leaching was solubility limited, the release curves would be expected to diverge with dissimilar total release at completion (Fig. 1). Furthermore, the measured concentrations of additives in the eluates were also compared to their respective water solubility limits. The concentrations of UV-234 and HAS-1 remained less than 10% of their respective solubility limits at all time points. The concentrations of AO-1076 and AO-1010 approached 50% of their solubility limit at the high solid to liquid ratio and later leaching time points (*t* = 32 to 64 d), while the concentration of AO-168 approached its solubility limit at the 64 d sampling time point. However, for these five additives (UV-234, HAS-1, AO-1076, AO-1010, and AO-168) total additive release at 64 d was not significantly different (p > 0.05) between the low, mid, and high solid to liquid ratios, indicating that sink conditions were maintained throughout the duration of the test. Leaching experiments for the PVC_DBP plastic were performed using lower solid to liquid ratios (10 to 250 mg per 50 mL) to account for the higher additive loading (21% DBP) in this plastic. At the high solid to liquid ratio (250 mg per 50 mL), the concentration of DBP in the eluates approached its solubility limit at all time points. The proportion of total DBP that leached into aqueous solution after 64 d at the high solid to liquid ratio (5.9%) was significantly less (p < 0.05) than that observed for either the mid (30.8%) or low (31.5%) solid to liquid ratios, clearly indicating that leaching at the high solid to liquid ratio was solubility limited. Given the substantially higher loading of DBP in PVC_DBP compared to additives in LDPE_AO, PET_UV, and PA6_HAS, this was in line with expectation. However, at the low and mid solid to liquid ratios sink conditions were maintained over the duration of the leaching test, with the concentration of DBP not exceeding more than 50% of its solubility limit. This example clearly demonstrates the benefit of dynamic leaching methods when simulating environmental compartments where dilution is likely to be high. Where sink conditions are suitably maintained, this method of assessing additive leaching from plastic provides a relevant approach for simulating the dynamics of additive leaching that can occur within oceans, rivers, lakes, and soil which effectively act as infinite sinks.

Reproducibility was assessed as the relative standard deviation of measured concentrations of additives in replicate samples (*n* = 3). In general, variance increased with decreasing analyte concentration (Fig. 1). For additives leaching from LDPE_AO the variance was highest at early sampling time points (up to 121% RSD) owing to the low analyte concentrations. The variability in the measurement of additive UV-234 in eluates was high (7 to 123% RSD), but improved at the high solid to liquid ratio (7 to 36% RSD), where analyte concentrations were comparatively higher. These observations reflect the additive concentrations being near the lower end of the method calibration range where they are approaching their respective LOQ. By comparison, reproducibility was markedly better for HAS-1 (4.3 to 10.7% RSD) and DBP (1.3 to 37.7% RSD), attributed to the higher analyte concentrations measured for each of these additives at all sampling times.

Recovery was evaluated by comparing leaching results with the gravimetrically determined loss in mass of the experimental plastics and quantification of residual additive in the experimental plastics at the completion of the experiment (Table 5). The gravimetric mass loss for PVC_DBP after 64 d (29.1%) was concordant with the cumulative additive release determined through the leaching experiment (30.8%) indicating excellent recovery. In contrast, the mass loss for PA6_HAS, LDPE_AO, and PET_UV was significantly greater than that calculated from cumulative additive release determined through the leaching experiment. However, the low proportion of additive in these plastics (< 0.5%) would have resulted in minimal mass loss from the cumulative release of additive into the media. Rather, the observed mass loss was attributed to the release of non-target compounds (see discussion below) and shedding of microplastic fragments which may be released and lost throughout the experiment during decanting and filtering steps (several fragments visually observed to collect on filter mesh during sampling procedure). Solvent extraction of PA6_HAS indicated a 55.7% loss of HAS-1, which was not significantly different to the measured cumulative proportion of HAS-1 released into the eluates over the 64 days (38.5%). However, the proportion of DBP released as determined by solvent extraction of the residual experimental plastic (18.6%) was not consistent with the cumulative proportion of DBP determined from the analysis of the eluates, or gravimetric mass loss. This observation may be due to incomplete solvent extraction of DBP [35].

**Table 5:** Evaluation of recovery by comparison of cumulative additive release at 64 d with mass loss and residual additive determined by solvent extraction.

Plastic	Cumulative additive release (%) 1	Mass loss (%) 1	Additive loss by solvent extraction (%) 1
PA6_HAS	38.5 ± 3.4	1530 ± 561	55.7 ± 9.9%
PVC_DBP 2	30.8 ± 2.4	29.1 ± 5.2	18.6 ± 4.8%
LDPE_AO	0.0317 ± 0.0031	41.6 ± 14.4	ND
PET_UV	2.66 × 10^−4^ ± 1.2 × 10^−4^	412 ± 239	ND

1 Expressed as percent of starting additive concentration;.

2 Calculated on low and mid solid to liquid ratios where sink conditions were shown to be maintained.

**Fig. 2:**
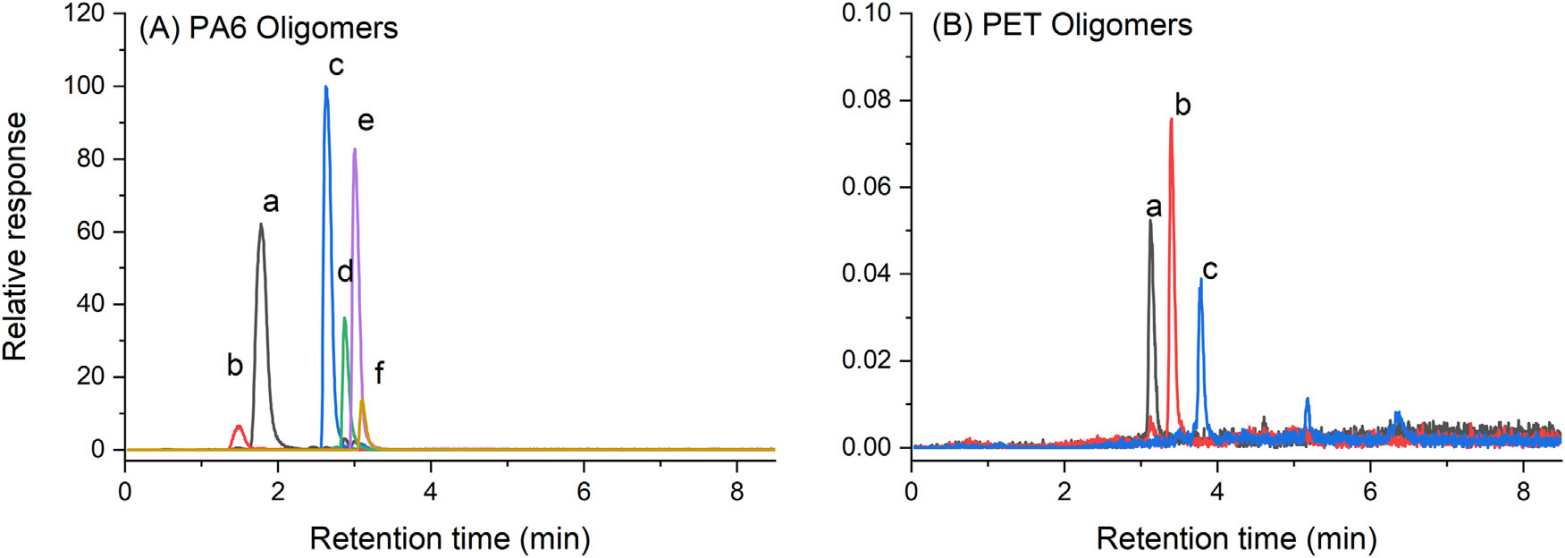
LC-MS extracted ion chromatograms showing oligomer peaks identified in (A) PA6_HAS 16 day eluate ((a) caprolactam, (b) PA6 dimer, (c) PA6 trimer, (d) PA6 tetramer, (e) PA6 pentamer, (f) PA6 hexamer) and (B) PET_UV 1 day eluate ((a) linear dimer S2, (b) cyclic dimer S2, (c) cyclic trimer S1).

Identification of non-target compounds was facilitated by the full scan high-resolution MS data obtained through the use of QTOF instrumentation. For PA6_HAS, residual monomer (caprolactam) and cyclic oligomers (dimer to hexamer) were tentatively identified in the eluates, with accurate mass and elution order consistent with previous reports (Fig. 2) [36,37]. Similarly, a linear dimer, cyclic dimer, and cyclic trimer residue were tentatively identified in PET_UV eluates, consistent with previous reports [38]. This demonstrated the utility of the LC-QTOF method for eluate analysis enabling both target compound quantification, but also non-target screening.

**Fig. 3:**
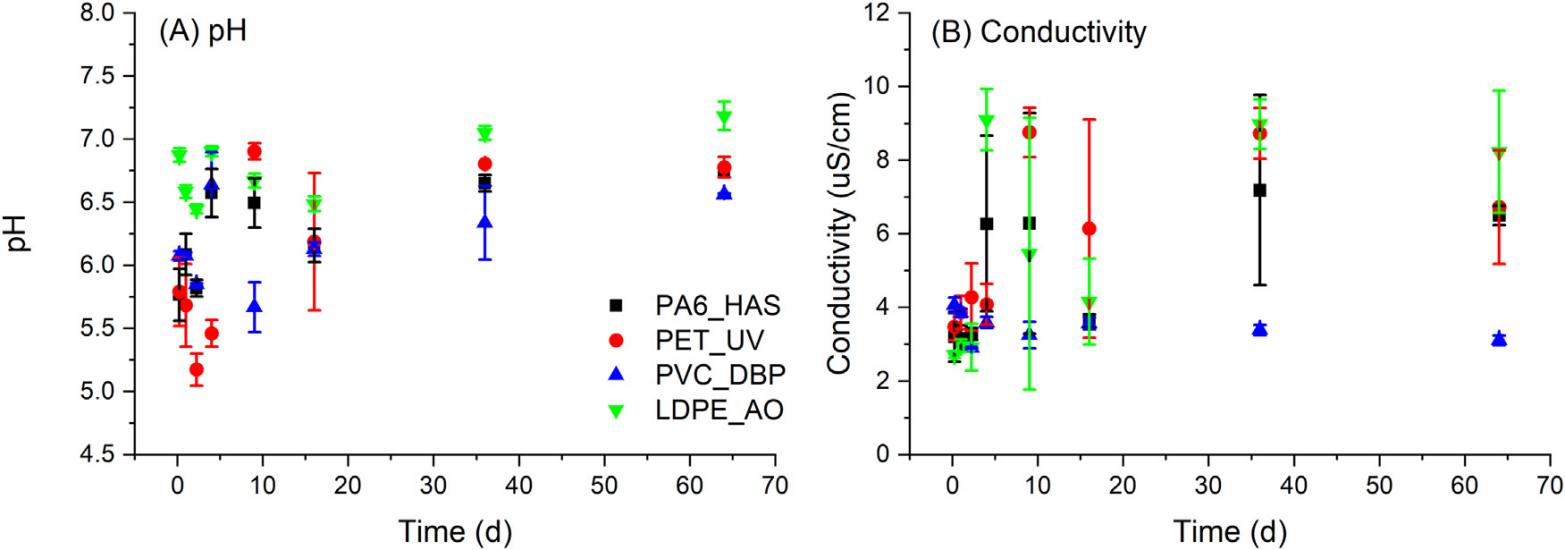
Non-specific analysis of eluates generated at mid solid to liquid ratio for all samples; (A) pH and (B) conductivity (error bars = standard deviation).

**Fig. 4:**
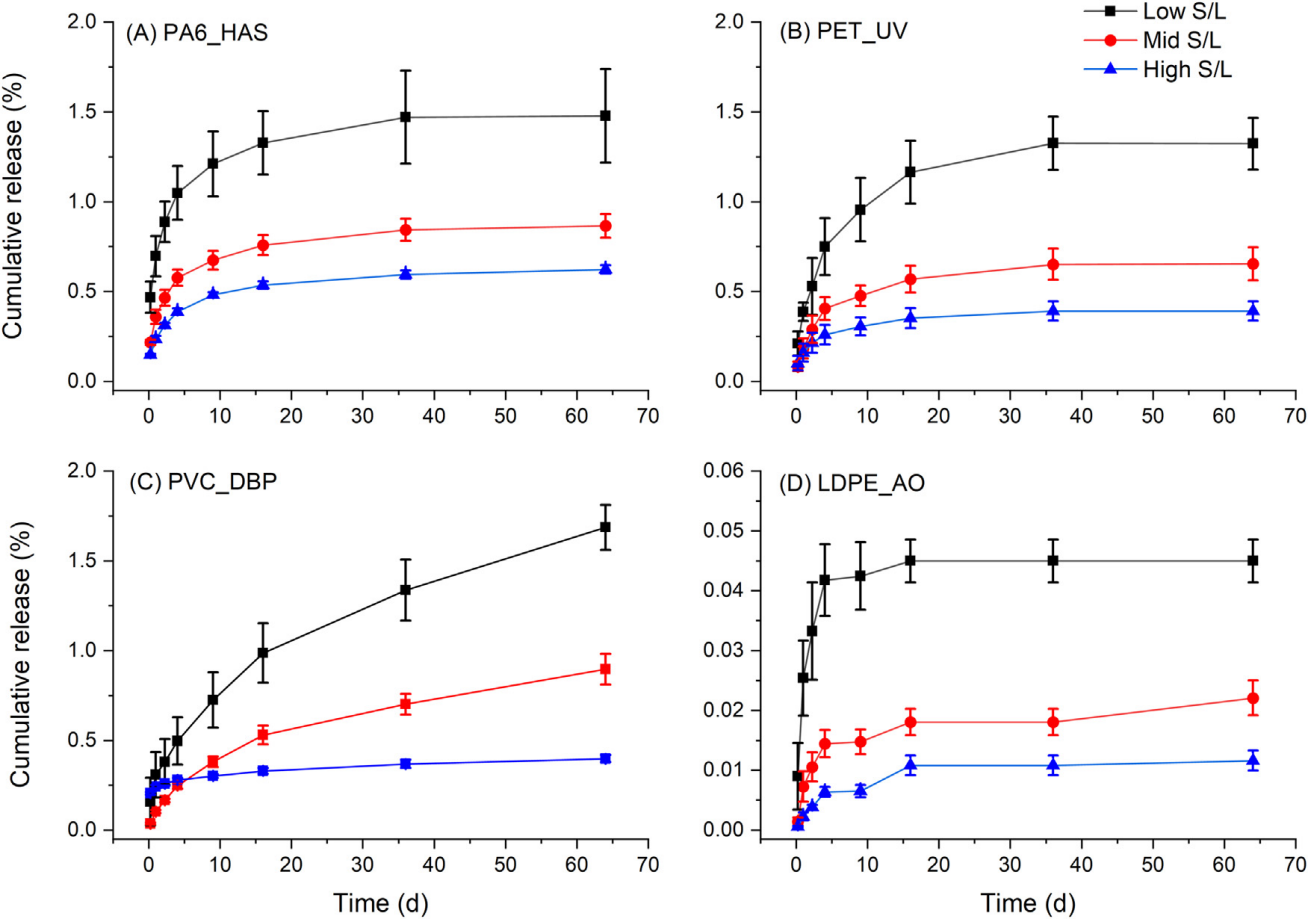
NPOC cumulative release for four different plastics (as a percent of total plastic mass) (error bars = standard deviation).

To enable further insights into the leaching processes, non-specific analyses were performed on the eluates, including pH, conductivity, and NPOC. Eluate pH varied between 6.6 to 7.1 for blank samples and 5.2 to 7.2 across the four plastic samples (Fig. 3). The pH of PA6_HAS and PET_UV eluates was approximately 1 unit less than the control at early time points (6 h to 2.25 d), with no difference observed at longer time points. PVC_DBP eluates generally exhibited pH lower than control for all time points, while no difference in pH was observed for LDPE_AO eluates. Conductivity varied between 1.9 to 9.7 µS for the blanks and 1.7 to 10.7 µS across the four plastic samples. Although, replicate variance was typically large with no trends identifiable across the course of the time series or between samples. NPOC measurements of the UP water blank samples indicated the presence of low levels of organic carbon up to 2.2 ppm at early sampling time points (6 h to 2.25 d) decreasing to less than the LOQ at all subsequent time points (4 d to 64 d). The presence of low levels of organic carbon at these initial sampling time points may be due to residue carbonaceous deposits on the glass bottles from the furnace cleaning procedure. By contrast, eluate solutions from the plastic samples exhibited NPOC concentrations of up to 31.2 ppm (Fig. 4), being significantly higher than that of the UP water blank samples. Variance between replicate samples was acceptable, typically less than 20% RSD. The cumulative percent NPOC varied with solid to liquid ratio for all plastics, which may indicate solubility limited release of an organic component not identified by LC-MS. Alternatively organic carbon may arise from biological growth leading to carbon assimilation independent of the additive leaching p process, however the near zero NPOC of blank samples indicated that biological growth was not occurring and contributing to organic carbon in the sample eluates.

### Quality assurance and analytical precautions

A number of precautions were adopted to minimise introducing contamination into the experimental system. All glassware was rinsed with propan-2-ol, acetone and UP water followed by baking of non-volumetric glassware at 450 °C for 8 h. Mobile phases were prepared gravimetrically to avoid contact with volumetric glassware such as measuring cylinders. Solvents used for mobile phase preparation were identified as a source of DBP, AO-1010, and AO-1076 contamination, evident as ghost peaks. Contamination with AO-1076 and AO-1010 was identified to originate from the solvent bottle cap after first opening and use. It was hypothesised that residual solvent on the bottle opening came into contact with the plastic cap when it was replaced after use, leading to extraction of additives from the cap during storage. When the bottle was subsequently opened and solvent decanted, the extracted plastic additives from the cap were washed off the bottle opening contaminating the mobile phase preparation. Placing aluminium foil over the bottle opening before replacing the plastic cap effectively minimised this source of contamination and was incorporated into the routine analytical procedure. For sample preparation, volumetric glassware (e.g. volumetric pipettes and syringes) is typically preferred over plastic consumables (e.g. capillary piston pipettes) to avoid potential contamination from extractable plastic additives and other chemical contaminants. However, to simplify the sample preparation workflow and decrease analysis time, capillary piston pipettes were evaluated as a more convenient alternative. Pipette tips were extracted with methanol through normal use and the resulting solvent compared by LC-MS analysis with that obtained for methanol transferred via a glass pipette. AO-1010 was the only target analyte identified to be extracted from the plastic capillary piston pipette tips. Other extraneous peaks in the chromatograms of methanol dispensed with plastic capillary piston pipette tips were tentatively identified from their mass spectra to be erucamide, glycerol monopalmitate, eicosenamide, and 1-stearoylglycerol (Fig. S3). These compounds are commonly used as lubricants, dispersants, antistatic agents, and release agents in plastic formulations [1]. As the retention times of these compounds were similar to those for UV-234 and DBP, their presence was concerning as they could potentially lead to significant to ion suppression of target additive chemicals or internal standards. Pre-rinsing of the plastic capillary piston pipette tips with five aliquots of acetone minimised both AO-1010 (Fig. S4) and non-target extractables to an extent where interference was negligible. Pre-rinsing of the plastic capillary piston pipette tips with acetone was subsequently incorporated into the routine analytical procedure.

These precautions are not exclusive to chemical based assessments of plastic additive leaching as described in this method and should also be considered when conducting biological assessments of chemicals. While the use of appropriate control treatments can be insightful in such studies, they do not take account of the potential for synergistic toxicity arising from exposure of test organisms to mixtures of contaminants. In this context the presence, and impact, of extraneous plastic additives or NIAS sourced from plastic materials used in experimental systems will be extremely difficult to identify and differentiate from the effect of a specific test substance. This is particularly relevant in modern testing laboratories where the use of plastic derived materials (e.g. microplates, multichannel pipettes, centrifuge tubes, syringe filters, and extraction systems) is firmly imbedded due to the convenience they provide and their relatively cheap costs of purchase.

## CRediT authorship contribution statement

**James H. Bridson:** Conceptualization, Methodology, Validation, Formal analysis, Investigation, Writing – original draft. **Robert Abbel:** Conceptualization, Methodology, Writing – review & editing. **Dawn A. Smith:** Conceptualization, Methodology, Writing – review & editing. **Grant L. Northcott:** Conceptualization, Writing – review & editing, Project administration, Funding acquisition. **Sally Gaw:** Conceptualization, Writing – review & editing, Supervision.

## Declaration of Competing Interest

The authors declare that they have no known competing financial interests or personal relationships that could have appeared to influence the work reported in this paper.

## Data Availability

Data will be made available on request.

## Appendix Supplementary materials

Image, application 1
