# Supplementary material for: Solving a microplastic dilemma? Evaluating additive release with a dynamic leaching method for microplastic assessment (DyLeMMA)
Source: MethodsX. 2023 May 19;10:102221. doi: 10.1016/j.mex.2023.102221 (PMC10225925; doi:10.1016/j.mex.2023.102221)
Supplement: Supplementary file 1 [file mmc1.pdf]

Supporting Information

Solving a microplastic dilemma? Evaluating additive release with a dynamic leaching method for microplastic assessment (DyLeMMA)

James H. Bridson [a, c] *, Robert Abbel [a], Dawn A. Smith [a], Grant L. Northcott [b] and Sally Gaw [c]

[a] Scion, Titokorangi Drive, Private Bag 3020, Rotorua 3046, New Zealand
[b] Northcott Research Consultants Limited, 20 River Oaks Place, Hamilton 3200, New Zealand
[c] School of Physical and Chemical Sciences, University of Canterbury, Christchurch 8041, New Zealand

*Corresponding author: jamie.bridson@scionresearch.com; +64 7 343 5899

Method S1. Assessment of different dynamic leaching approaches

Three different approaches were assessed to determine the leaching of additive from plastic into aqueous media, being: (i) immiscible organic solvent sink, (ii) a solid phase sink, and (iii) sequential media replenishment. For the first two methods the partitioning rate from the aqueous phase to the sink material was first evaluated followed by a proof of concept leaching experiment to evaluate the method.

(i) Biphasic infinite sink method

The partitioning rate of additives from the aqueous phase to the octanol phase was evaluated using spiked solutions prepared by adding 100 µg of AO-1076, AO-168 or UV-234 as a 1 mg/mL solution in methanol into 50 mL of water. n-Octanol (10 mL) was added, the bottle capped and incubated at room temperature with agitation at 100 rpm overnight. An aliquot of n-octanol (100 µL) was withdrawn at 5, 15, 30, 60, 120, 360, and 1320 min. An aliquot of water (1 mL) was also withdrawn at 60 and 1320 min for analysis. Leaching experiments were performed by cutting plastics into microplastic sized pieces (1-5 mm) and placing ca. 500 mg into a 50 mL bottle and adding 40 mL of water followed by 10 mL of n-octanol. The bottles were incubated at varying temperatures (25 to 40 °C) with orbital shaking (100 rpm) for a period of up to 37 d. n-Octanol (100 µL) and/or water (1 mL) was withdrawn at various time points and stored at 4 °C. Octan-1-ol samples were diluted 10 µL (plus internal standard) to 1000 µL in methanol for analysis.

(ii) Solid phase infinite sink method

The partitioning rate was assessed using spiked solutions as described above. SPE discs were pre-conditioned according to the manufacturer's instructions. Briefly, SPE discs were washed with acetone (20-30 mL), methanol (20-30 mL) and finally water (20-30 mL) before cutting into quarters and storing in water. One piece of SPE disc (C18, HLB, or SDB-RPS) was placed into a bottle containing water, spiked with Irganox 1076 or UV-234, and incubated at room temperature with agitation at 120 rpm for 93 h. The SPE discs were withdrawn at 0.25, 1, 4, 49, and 93 h and dried under vacuum at 40 °C. The SPE discs were back extracted three times by soaking with 5 mL of acetone for 10 min with the extracts pooled and blown to dryness under nitrogen. Samples were re-dissolved in 1 mL of methanol and diluted 10 µL (plus internal standard) to 1000 µL in methanol for analysis. Extraction efficiency was validated by repeating back extraction a further two times for a select number of samples. Leaching experiments were performed by cutting plastics (AIM2 deployment plastics) into microplastic sized pieces (1-5 mm) and placing 500 mg into a bottle with 50 mL of water. One piece of preconditioned SPE disc (HLB) was placed into the bottle which

was incubated at 30 °C with orbital shaking (120 rpm) for up to 14 d. The SPE discs were withdrawn and replaced at 4, 24, 48, 120, 192, and 336 h, rinsed with water and dried under vacuum at 40 °C. At 48 and 336 h time points, 5 mL of water was withdrawn and evaporated to dryness under vacuum. SPE discs were back extracted as described above for analysis. Water samples were dissolved in 1 mL of methanol with internal standard for analysis.

(iii) Sequential batch method

The water solubility of each additive was evaluated by adding c.a. 0.5 mg of additive into 15 mL of water and incubating at 30 °C with orbital shaking (120 rpm) for 48 h before allowing to cool to room temperature for a further 24 h. The water was filtered through a 13 mm nylon 0.22 µm syringe filter for analysis by LC-MS. Leaching experiments were performed by cutting plastics (AIM2 deployment plastics) into microplastic sized pieces (1-2 mm) and placing 500 mg into a 100 mL bottle with 50 mL of water. The bottles were incubated at 30 °C with orbital shaking (120 rpm) for a period of up to 16 d. At each sampling time point (0.2, 1, 2, 6, 11, and 16 d) the water phase was decanted through stainless steel mesh (420 µm) into glass bottles (stored at 4 °C), replaced with a further 50 mL of water and returned to the incubator. Samples were concentrated using a SP Genevac EZ-2 Plus centrifugal evaporator (Pennsylvania) by transferring 2.4 mL of water (plus internal standard) into a high recovery LC vial and evaporating to dryness at 45 °C under a nitrogen purge. Samples were made up with 100 µL of methanol for analysis by LC-MS. Recovery was evaluated by spiking at 1, 10 or 100 ng/mL analyte concentration and concentrating to dryness using the centrifugal evaporator as described above.

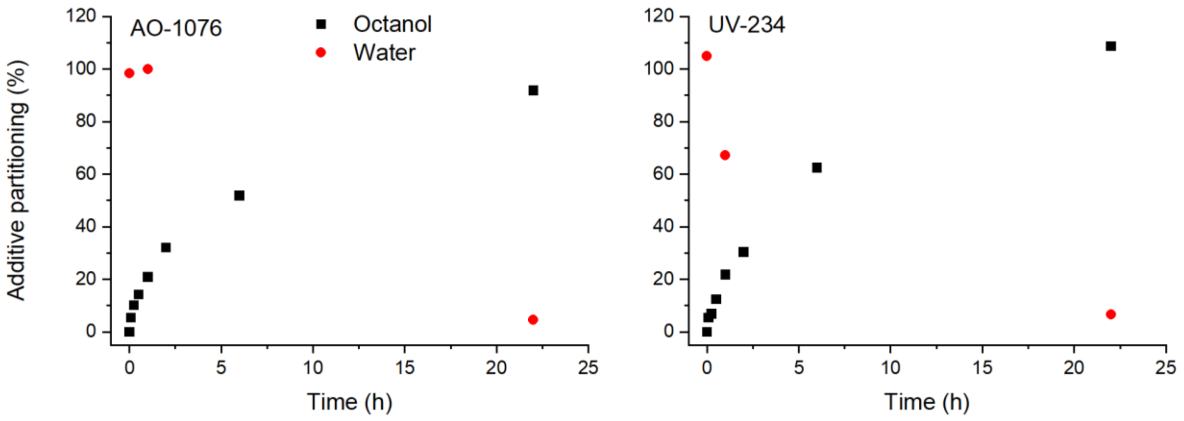

Figure S1. Partitioning profiles for AO-1076 and UV-234 from water to n-octanol phase.

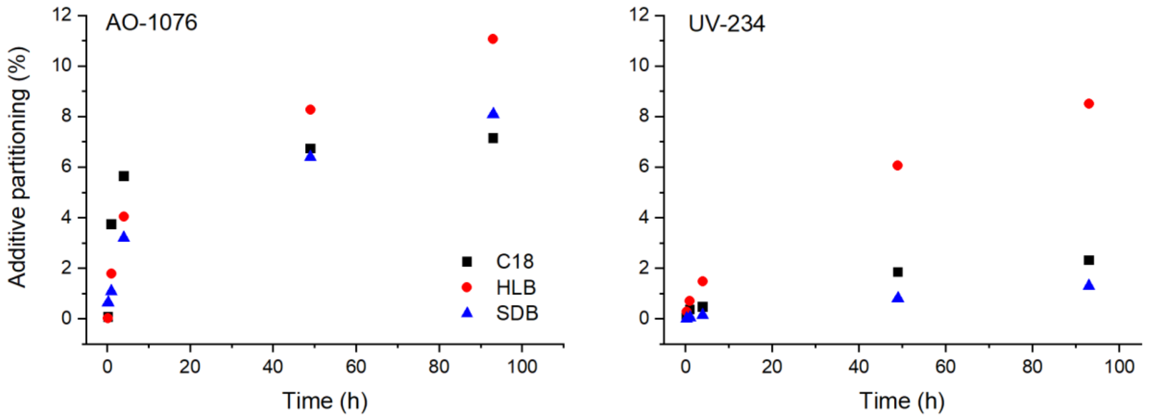

Figure S2. Partitioning profiles for AO-1076 and UV-234 from water onto three different SPE discs (C18, HLB, SDB-RPS).

Table S1. Comparison of additive solubility in water with maximum observed concentration for sequential batch leaching proof-of-concept experiment and method validation

Sample	Additive	Water solubility at 20 °C	Max concentration method development	Max concentration method validation
LDPE_AO	AO-1076	2.85 ng/mL [1]	0.05 ng/mL	1.4 ng/mL
	AO-1010	1.31 ng/mL [2]	0.13 ng/mL	0.83 ng/mL
	AO-168	1.4 ng/mL [3]	0.63 ng/mL	1.2 ng/mL
PET_UV	UV-234	0.25 ng/mL [2]	0.02 ng/mL	0.024 ng/mL
PA6_HAS	HAS-1	139 µg/mL [1]	ND	5.5 µg/mL
PVC_DBP	DBP	11.4 µg/mL [1]	ND	9.1 µg/mL

[1] Data from European Chemicals Agency database; [2] Determined experimentally; [3] Data from EPA ToxComp database

Table S2. Advantages and disadvantages of the three different leaching approaches evaluated

Method	Advantages	Disadvantages
(i) Immiscible organic solvent sink method	Easy and fast allowing high throughput<br>Rapid partitioning from water to n-octanol provides sink conditions<br>Cost-effective chemicals and consumables	Immiscible organic solvent is a potential source of contamination<br>Difficulty in concentrating octanol<br>Dissolved octanol may interfere with release from plastic
(ii) Solid phase (SPE) sink method	Inherent sample concentration provides good method sensitivity<br>Contamination easier to control than for biphasic infinite sink method<br>SPE disc provide sink conditions	Costly consumables<br>Labour intensive conditioning and back extraction steps<br>Multiple handling steps (e.g. conditioning and back extracting discs) potential for contamination<br>Partitioning rate from aqueous phase to SPE disc may be slower than release rate<br>Need to tailor SPE chemistry to target analytes
(iii) Sequential batch leaching method	Easy and fast allowing high throughput<br>Avoids contamination from infinite sink<br>Cost-effective chemicals and consumables<br>Avoids additional complication of partitioning rate<br>Allows other non-specific testing (e.g. NPOC, conductivity etc)	Necessity to check concentration is less than solubility limit and adjust solid to liquid ratio if required<br>Requires sample concentration to achieve sensitivity

Table S3. Analyte recovery from aqueous solution spiked with target additives after filtration using different syringe filters or filter paper

Analyte	Recovery (%) [1]					
	PA	PTFE	PVDF	PES	GFA	CA
AO-1076	0	12	36	124	0	0
AO-1010	0	0	46	24	11	0
AO-168	0	0	5	10	0	143
UV-234	1	0	36	16	5	1
DBP	150	81	107	289	0	138
HAS-1	93	39	0	0	ND	ND

[1] PA = 0.2 µm nylon syringe filter; PTFE = 0.2 µm polytetrafluoroethylene syringe filter; PVDF = 0.2 µm polyvinylidene fluoride syringe filter; PES = 0.45 µm polyethersulfone syringe filter; GFA = GF/A grade glass fibre filter paper; CA = 0.45 µm cellulose acetate filter membrane.

Table S4. Optimum MS interface parameters for each target analyte

Analyte	Optimum parameter setting (V)		
	Capillary voltage	Nozzle voltage	Fragmentor voltage
AO-1076	3000-4000	500	200
AO-1010	4000	500	225
AO-168	3000-4000	500	200
UV-234	3000	500	175
DBP	3500	0	125
HAS-1	3000-4000	0	125

Table S5. Evaluation of different weightings applied to least squares regression for each analyte

Weighting	AO-1076		AO-1010		AO-168		UV-234		DBP		HAS-1	
	R^2	%RA	R^2	%RA	R^2	%RA	R^2	%RA	R^2	%RA	R^2	%RA
None	0.997	56.7	0.997	55.6	0.998	61.9	0.996	54.3	0.998	90.1	1.000	80.2
1/x	0.996	91.8	0.996	91.6	0.998	90.6	0.995	90.3	0.995	90.3	0.995	90.8
$1/x^2$	0.993	94.6	0.993	94.6	0.985	91.5	0.996	90.5	0.970	90.1	0.955	84.4
1/y	0.996	90.9	0.996	91.6	0.998	89.5	0.995	90.2	0.995	85.3	0.994	90.6
$1/y^2$	0.995	94.5	0.993	94.5	0.986	91.0	0.995	90.1	0.983	89.2	0.950	84.0
$1/SD^2$	0.999	92.2	0.996	94.6	0.994	89.1	0.998	91.5	0.989	89.6	0.995	87.5

R^2 = correlation coefficient; %RA = percent residual accuracy.

Table S6. Non-purgeable organic carbon (NPOC) method validation parameters

Parameter	Range	R^2	LOD	LOQ	Carryover (25 to 0 ppm)	Accuracy (at 25 ppm)	Precision (at 25 ppm)
NPOC	0-100 ppm	1.000	0.06 ppm	0.21 ppm	< 0.21 ppm	92 %	1.2 %

R^2 = correlation coefficient; LOD = limit of detection; LOQ = limit of quantification.

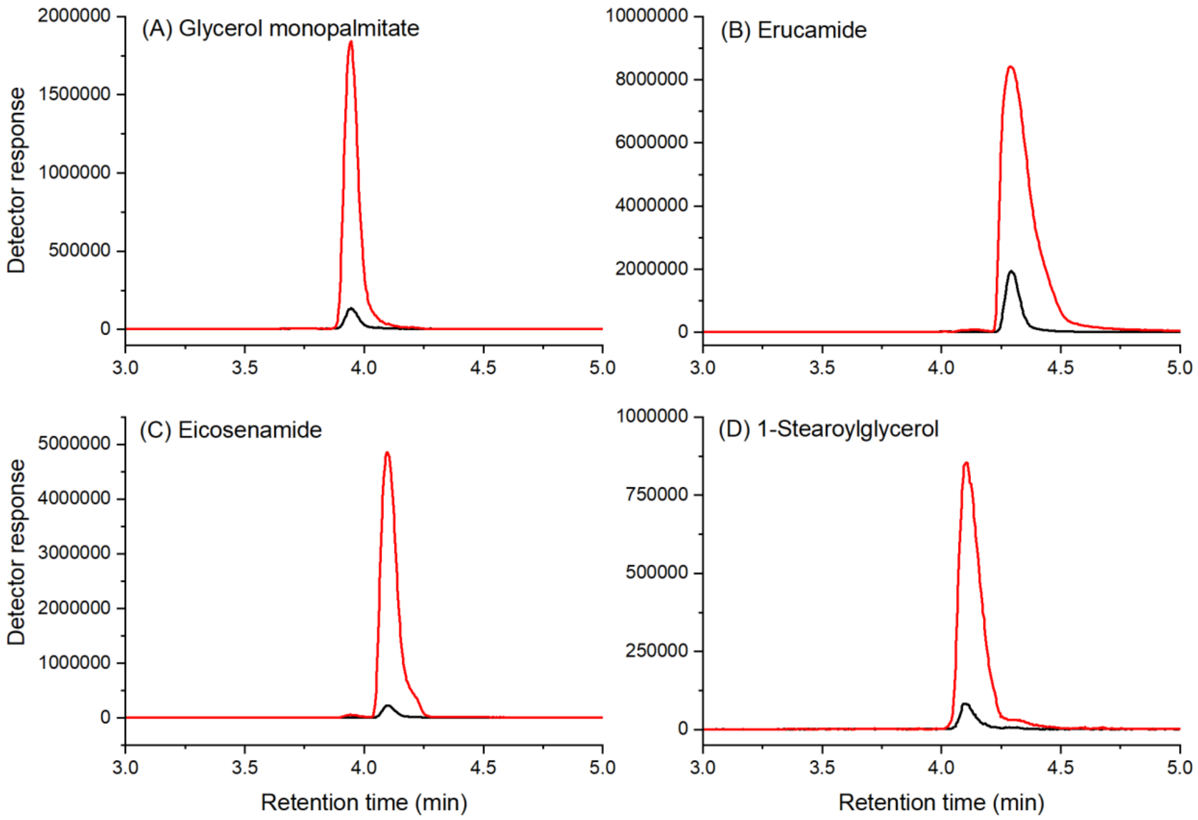

Figure S3. Extracted ion chromatogram for capillary piston pipette tip methanol extractables before (red trace) and after rinsing with acetone (black trace).

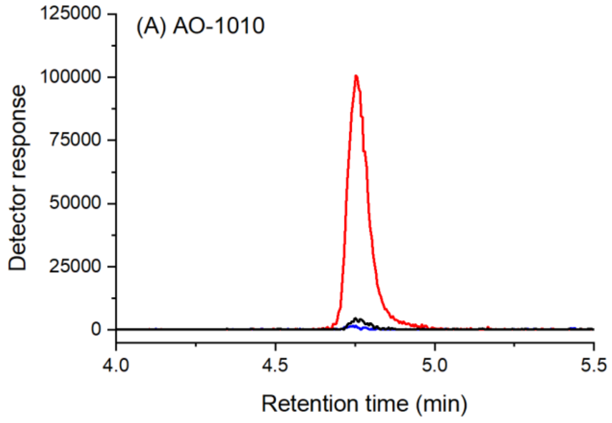

Figure S4. AO-1010 extracted ion chromatogram for capillary piston pipette tip methanol extractables before (red trace) and after rinsing with acetone (black trace) and compared with a blank injection (blue trace).
